# Supplementary material for: Transcriptome Analysis Reveals Vimentin-Induced Disruption of Cell–Cell Associations Augments Breast Cancer Cell Migration
Source: Cells. 2022 Dec 13;11(24):4035. doi: 10.3390/cells11244035 (PMC9776984; doi:10.3390/cells11244035)
Supplement: Supplementary file 1 [file cells-11-04035-s001.zip › DEGs and enrichment analysis.zip!/DEGs and enrichment analysis/WT_VIMvsVector GO enrichment/GSEA/WT_VIM/COLLAGEN_BIOSYNTHETIC_PROCESS/COLLAGEN_BIOSYNTHETIC_PROCESS(GO_0032964).html]

Details for gene set COLLAGEN\_BIOSYNTHETIC\_PROCESS(GO:0032964)[GSEA]


|  || Dataset | fpkm.sample |
| Phenotype | sample.cls |
| Upregulated in class | WT\_VIM |
| GeneSet | COLLAGEN\_BIOSYNTHETIC\_PROCESS(GO:0032964) |
| Enrichment Score (ES) | 0.9351388 |
| Normalized Enrichment Score (NES) | 1.4723406 |
| Nominal p-value | 0.0 |
| FDR q-value | 0.16299988 |
| FWER p-Value | 0.163 |
Table: GSEA Results Summary

  

Fig 1: Enrichment plot: COLLAGEN\_BIOSYNTHETIC\_PROCESS(GO:0032964)
  
Profile of the Running ES Score & Positions of GeneSet Members on the Rank Ordered List

  

| PROBE | DESCRIPTION   (from dataset) | GENE SYMBOL | GENE\_TITLE | RANK IN GENE LIST | RANK METRIC SCORE | RUNNING ES | CORE ENRICHMENT || 1 | ENSG00000026025 | VIM |  |  | 2388 | 1709.539 | 0.9351 | Yes |
| 2 | ENSG00000180210 | F2 |  |  | 3166 | 4.318 | 0.9245 | No |
| 3 | ENSG00000163501 | IHH |  |  | 3524 | 3.632 | 0.9205 | No |
| 4 | ENSG00000118513 | MYB |  |  | 3718 | 3.349 | 0.9191 | No |
| 5 | ENSG00000110811 | P3H3 |  |  | 5219 | 2.310 | 0.8951 | No |
| 6 | ENSG00000125378 | BMP4 |  |  | 6326 | 1.969 | 0.8775 | No |
| 7 | ENSG00000141696 | P3H4 |  |  | 6433 | 1.937 | 0.8768 | No |
| 8 | ENSG00000119699 | TGFB3 |  |  | 7011 | 1.815 | 0.8681 | No |
| 9 | ENSG00000108821 | COL1A1 |  |  | 7546 | 1.706 | 0.8600 | No |
| 10 | ENSG00000105329 | TGFB1 |  |  | 8097 | 1.609 | 0.8516 | No |
| 11 | ENSG00000106991 | ENG |  |  | 9036 | 1.476 | 0.8366 | No |
| 12 | ENSG00000161544 | CYGB |  |  | 9565 | 1.409 | 0.8284 | No |
| 13 | ENSG00000141570 | CBX8 |  |  | 10184 | 1.336 | 0.8187 | No |
| 14 | ENSG00000112033 | PPARD |  |  | 12078 | 1.175 | 0.7874 | No |
| 15 | ENSG00000149257 | SERPINH1 |  |  | 12189 | 1.166 | 0.7862 | No |
| 16 | ENSG00000260428 | SCX |  |  | 12314 | 1.156 | 0.7847 | No |
| 17 | ENSG00000179583 | CIITA |  |  | 13038 | 1.099 | 0.7731 | No |
| 18 | ENSG00000162552 | WNT4 |  |  | 13603 | 1.056 | 0.7642 | No |
| 19 | ENSG00000163794 | UCN |  |  | 14031 | 1.023 | 0.7575 | No |
| 20 | ENSG00000196591 | HDAC2 |  |  | 16306 | 0.899 | 0.7196 | No |
| 21 | ENSG00000065308 | TRAM2 |  |  | 17574 | 0.823 | 0.6986 | No |
| 22 | ENSG00000094975 | SUCO |  |  | 17899 | 0.804 | 0.6936 | No |
| 23 | ENSG00000156140 | ADAMTS3 |  |  | 18654 | 0.762 | 0.6812 | No |
| 24 | ENSG00000166173 | LARP6 |  |  | 18785 | 0.754 | 0.6795 | No |
| 25 | ENSG00000167711 | SERPINF2 |  |  | 19629 | 0.709 | 0.6656 | No |
| 26 | ENSG00000181104 | F2R |  |  | 19822 | 0.698 | 0.6628 | No |
| 27 | ENSG00000116473 | RAP1A |  |  | 20934 | 0.638 | 0.6443 | No |
| 28 | ENSG00000113721 | PDGFRB |  |  | 23668 | 0.464 | 0.5983 | No |
| 29 | ENSG00000102760 | RGCC |  |  | 23769 | 0.459 | 0.5969 | No |
| 30 | ENSG00000164171 | ITGA2 |  |  | 24470 | 0.402 | 0.5853 | No |
| 31 | ENSG00000130635 | COL5A1 |  |  | 24559 | 0.394 | 0.5840 | No |
| 32 | ENSG00000132170 | PPARG |  |  | 25521 | 0.307 | 0.5679 | No |
| 33 | ENSG00000116285 | ERRFI1 |  |  | 25692 | 0.284 | 0.5652 | No |
| 34 | ENSG00000136244 | IL6 |  |  | 25778 | 0.273 | 0.5639 | No |
| 35 | ENSG00000118520 | ARG1 |  |  | 26099 | 0.240 | 0.5587 | No |
| 36 | ENSG00000118523 | CTGF |  |  | 26374 | 0.204 | 0.5541 | No |
| 37 | ENSG00000157613 | CREB3L1 |  |  | 27029 | 0.105 | 0.5431 | No |
| 38 | ENSG00000276365 | MIR145 |  |  | 29296 | 0.000 | 0.5048 | No |
| 39 | ENSG00000166396 | SERPINB7 |  |  | 29870 | 0.000 | 0.4951 | No |
| 40 | ENSG00000207739 | MIR218-2 |  |  | 32359 | NaN | 0.4530 | No |
| 41 | ENSG00000207732 | MIR218-1 |  |  | 32364 | NaN | 0.4529 | No |
| 42 | ENSG00000283797 | MIR29B1 |  |  | 32977 | NaN | 0.4426 | No |
| 43 | ENSG00000283705 | MIR92A1 |  |  | 38450 | NaN | 0.3500 | No |
| 44 | ENSG00000284203 | MIR29B2 |  |  | 42617 | NaN | 0.2795 | No |
| 45 | ENSG00000284538 | MIR92A2 |  |  | 42833 | NaN | 0.2758 | No |
| 46 | ENSG00000125363 | AMELX |  |  | 44700 | NaN | 0.2443 | No |
| 47 | ENSG00000284032 | MIR29A |  |  | 45644 | NaN | 0.2283 | No |
Table: GSEA details
[plain text format]

  

Fig 2: COLLAGEN\_BIOSYNTHETIC\_PROCESS(GO:0032964)
  
Blue-Pink O' Gram in the Space of the Analyzed GeneSet

  

Fig 3: COLLAGEN\_BIOSYNTHETIC\_PROCESS(GO:0032964): Random ES distribution
  
Gene set null distribution of ES for
 **COLLAGEN\_BIOSYNTHETIC\_PROCESS(GO:0032964)**
